# Results of combined treatment of anaplastic thyroid carcinoma (ATC)

**DOI:** 10.1186/1471-2407-11-469

**Published:** 2011-11-01

**Authors:** Olfa Derbel, Sami Limem, Céline Ségura-Ferlay, Jean-Christophe Lifante, Christian Carrie, Jean-Louis Peix, Françoise Borson-Chazot, Claire Bournaud, Jean-Pierre Droz, Christelle de la Fouchardière

**Affiliations:** 1 University of Lyon - Leon-Berard Cancer Center, Department of Medical Oncology, 28 rue Laennec, 69003 Lyon, France; 2 Department of General and Endocrine Surgery, University Hospital Lyon-Sud, 165 chemin du Grand Revoyet, 69495 Pierre-Bénite, France; 3 Department of Nuclear Medicine, University Hospital Louis Pradel, 28 Avenue Doyen Lépine, 69500 Bron, France

## Abstract

**Background:**

Anaplastic thyroid carcinoma (ATC) is among the most aggressive human malignancies. It is associated with a high rate of local recurrence and with poor prognosis.

**Methods:**

We retrospectively reviewed 44 consecutive patients treated between 1996 and 2010 at Leon Berard Cancer Centre, Lyon, France. The combined treatment strategy derived from the one developed at the Institut Gustave Roussy included total thyroidectomy and cervical lymph-node dissection, when feasible, combined with 2 cycles of doxorubicin (60 mg/m2) and cisplatin (100 mg/m2) Q3W, hyperfractionated (1.2 Gy twice daily) radiation to the neck and upper mediastinum (46-50 Gy), and then four cycles of doxorubicin-cisplatin.

**Results:**

Thirty-five patients received the three-phase combined treatment. Complete response after treatment was achieved in 14/44 patients (31.8%). Eight patients had a partial response (18.2%). Twenty-two (50%) had progressive disease. All patients with metastases at diagnosis died shortly afterwards. Thirteen patients are still alive. The median survival of the entire population was 8 months.

**Conclusion:**

Despite the ultimately dismal prognosis of ATC, multimodality treatment significantly improves local control and appears to afford long-term survival in some patients. There is active ongoing research, and results obtained with new targeted systemic treatment appear encouraging.

## Background

Anaplastic thyroid carcinoma is an uncommon malignancy that accounts for only 2 to 5% of all thyroid cancers. It is one of the most aggressive human malignant tumors, in contrast to differentiated thyroid malignancies. Patients are typically elderly, with the majority older than 60 years [1,2]. At the time of diagnosis, approximately 40% of patients have distant metastases, 80% of them in the lung. Despite different treatment approaches, ATC grows rapidly, invades adjacent tissues, and most patients die due to uncontrolled local tumor invasion causing suffocation [3,4]. The treatment options for ATC include surgery, chemotherapy and radiotherapy, but all of these, especially if used alone, generally fail to control local disease.

Multimodal therapy combining surgery, chemotherapy and radiation therapy, can achieve better results in avoiding death from local invasion and suffocation and improving survival in some patients [5,6]. Nevertheless, the aggressive nature and rarity of ATC make it difficult to compare patient outcomes, especially in studies with small cohorts and short follow-up. A standardized successful protocol remains to be established and the optimal sequence of multimodal therapy is still debated [7]. In France, all cancer centers treating patients with anaplastic thyroid carcinomas use a standard treatment called the "IGR protocol" named after the Institut Gustave Roussy where it was proposed and first published [6].

Here, we retrospectively report the clinical outcome of all ATC patients treated in our Institution between 1996 and 2010.

## Methods

The clinical records of all patients with anaplastic thyroid carcinoma referred to the Centre Leon Berard between 1996 and 2010 were reviewed. Data were extracted for all patients with a confirmed diagnosis of anaplastic thyroid cancer.

Diagnosis was established on the basis of histological or cytological (fine-needle biopsy) features and was confirmed, when necessary, by immunochemical staining.

All patients underwent computed tomography (CT) of the neck and chest before or after thyroid surgery. Tumor staging was determined according to the TNM classification proposed by the American Joint Committee on Cancer (7th edition of TNM AJCC)[8]. "T" describes the size and location of the tumor. All anaplastic carcinomas are considered T4 tumors. T4a is for intrathyroidal anaplastic carcinomas--surgically resectable. T4b is for extrathyroidal anaplastic carcinomas--surgically unresectable. "N" refers to regional lymph node involvement (central, lateral cervical, and upper compartment).

No corresponds to no regional lymph node metastasis. N1, used to denote regional lymph node metastases, subdivides into N1a indicating metastases to level VI (pretracheal, paratracheal, and prelaryngeal lymph nodes), and N1b indicating unilateral or bilateral metastases to cervical or superior mediastinal lymph nodes. M1 is used to designate distant metastases. For thyroid cancer, this staging system differs with the tumor cell type: all anaplastic carcinomas are considered stage IV.

Surgical removal of the thyroid and cervical nodes, when possible, was generally the first step of the treatment. The surgical procedure was identified as total thyroidectomy, near total Thyroidectomy, biopsy or debulking. Total thyroidectomy denoted the removal of both lobes and the isthmus. Resection of one entire lobe, the isthmus and a portion of the other lobe was considered near total thyroidectomy. Any surgery less than resection of an entire lobe was considered a biopsy. Debulking was defined as a tumor reduction of more than 90%. The presence of residual tumor after surgery was evaluated by using the "R" classification with R0 corresponding to no residual tumor, R1 to microscopic residual tumor and R2 to macroscopic residual tumor. Radiotherapy (RT) was administered postoperatively. Hyperfractionated (two fractions of 1.2 Gy twice daily) or conventional radiation was used. Conventional radiotherapy was used instead hyper fractionated radiotherapy in poor general status patients. The RT volume was confined to the whole anterior half of the neck and upper 5 cm of the mediastinum. Radiation was achieved using two opposed antero-posterior fields. The total dose was estimated to vary from 46 to 50 Gy and was delivered over about 5 weeks. Concerning chemotherapy, two drugs, doxorubicin (50-60 mg/m2/d) and cisplatin (100 mg/m2/d) were used as a one-day treatment, repeated every 3 weeks.

Two cycles were administered before irradiation and 4 cycles afterwards. The time interval between chemotherapy and beginning/end of radiotherapy was 10 days. Carboplatine was used to replace cisplatin in combination with doxorubicin for patients with renal insufficiency.

Patients received GCSF and some had EPO primary prophylaxis. They also had nutritional support during radiotherapy. Evaluation of tumor response was performed after the first two courses of chemotherapy, after thyroid irradiation, one month after the end of treatment, then every three months. Tumor measurement techniques included physical examination and CT scan. The Response Evaluation Criteria in Solid Tumors (RECIST) criteria were used to assess response. Complete remission (CR) is defined as the complete disappearance of all clinically and radiographically demonstrable tumors and partial remission (PR) as a 30% decrease in the sum of the largest diameters of all measurable lesions. Progressive disease is defined as an increase of at least 20% in the sum of the largest diameters of all measurable lesions. Stable disease is the absence of CR or PR without any disease progression. Survival was measured from initiation of treatment. Kaplan Meier was used for statistical analysis.

The prognostic factors relatives to the patient were WHO performance status, age, sex, maximum diameter of tumor ≥ 5 cm, existence of acute symptoms at diagnosis, distant metastases, tracheal infiltration and WBC count (≥10,000/mm3). Impact of the treatment (type of surgery, residual disease after surgery, type of radiotherapy and chemotherapy, complete treatment) was also studied. Toxicity was evaluated in accordance with the National Cancer Institute Common Toxicity Criteria (NCI-CTC).

## Results

### Patient characteristics

During the time interval considered, 44 patients were referred to our institution (table 1). The majority were women (1.4:1). The age range was 44-80 years. A clinical history of pre-existing thyroid disease (thyroid nodules or goiter) was found in 10 patients (22%). The coincidental presence of a well-differentiated thyroid carcinoma was noted on examination of surgically resected ATC in 8 patients. Five of these had papillary carcinoma and 3 had follicular carcinoma.

**Table 1:** Patient characteristics.

Number of patients (%)	44
**Median age at diagnosis **(years)	65 (44-80)

**Gender**	
Female	26 (59)
Male	18 (41)
Female/male ratio	1.4

**Preexisting thyroid disease**	
Nodular goiter	10 (22.7)
Well-differentiated thyroid carcinoma	8 (18.1)

**Clinical presentation**	
Enlarged thyroid mass	38 (86.3)
Signs of local compression (dyspnea, dysphagia, dysphonia, and cervical pain).	32 (72.7)
Weight loss	12 (27.2)

**Clinical extent of disease**	
Local (T4 N0 M0)	12 (27.2)
Regional (T4 N1 M0)	12 (27.2)
Distant (T4 N0 M1, T4 N1 M1)	20 (45.4)

**Histology**	
Giant cells	19 (43.1)
Spindle cells	14 (31.8)
Mixed pattern	6 (13.6)
Not available	5 (11.3)

Thirty-eight patients (84%) presented with complaints of a sudden increase of a thyroid mass, 32 (71%) with signs of local compression (dyspnea, dysphagia, dysphonia, and cervical pain).

Twelve patients (26%) reported a weight loss. The WHO performance status was 0 or 1 for 38 patients and ≥2 for 6 patients. Clinicopathological factors relatives to patients are presented in tables 1.

### Tumour characteristics

Different morphological patterns of ATC were identified: giant, spindle or squamoid cell carcinomas, sometimes with a mixed pattern [8]. Large-cell and mixed pattern ATC were the most commonly observed pathological patterns. At diagnosis, tracheal infiltration was observed in 13 patients (30%). Thirty-nine patients presented with a tumor ≥ 5 cm. Distant metastases, most commonly located in the lungs, were found at the time of diagnosis in 21 patients, and 11 had more than 3 factors of poor prognosis.

### Treatment

The treatments are described in table 2.

**Table 2:** Treatment

Type of treatment (%)	
Surgery alone	2 (4.5)
Surgery + CT	3 (7)
Surgery + RT + CT	35 (79.5)
RT alone	2 (4.5)
Surgery + RT	2 (4.5)
**Type of surgery**	
Total thyroidectomy	28 (63.6)
Near total thyroidectomy	7 (15.9)
Biopsy/Debulking	9 (20.4)

**Residual disease after surgery**	
None	8 (18.1)
Microscopic residual disease	9 (20.4)
Macroscopic residual disease	25 (56.8)

**Type of radiotherapy**	
Conventional	5 (11.3)
Hyperfractionated	34 (77.2)

**Chemotherapy schedule**	
Doxorubicin+ cisplatin	33 (75)
Doxorubicin +carboplatin	3 (7)
Doxorubicin	1 (2.2)
Paclitaxel	1 (2.2)

**Treatment response**	
Complete response	14 (31.8)
Partial response	8 (18.1)
Disease progression	22 (50)

#### Surgery

Of the 44 patients studied, 28 had a total thyroidectomy, 7 had a near total thyroidectomy and 9 had debulking surgery. Tumor resection was complete in only 8 patients (18.2%) whereas 9 (20.5%) had microscopic residual disease (resection R1) and 25 (56.8%) macroscopic residual disease (R2). Lymph node involvement was found in 22 patients. Nineteen patients had node resection. After surgery, 10 patients encountered unilateral vocal cord paralysis, 4 patients had hemorrhage in surgical site and 2 patients presented hypocalcaemia despite of calcium supplement. No patient required tracheotomy during treatment.

#### Post-operative treatment

Thirty-nine patients received radiation to the thyroid area. Hyperfractionated radiotherapy was used in 34 patients whereas 5 had conventional radiation.

Thirty-eight patients (80%) received chemotherapy; thirty-three received a combination of doxorubicin and cisplatin, three received doxorubicin and carboplatin, one received doxorubicin alone and one received paclitaxel. Among the 35 patients who received the three-phase combined treatment, 14 (41%) received the full six scheduled cycles of chemotherapy. Treatment was stopped because of toxicity in 4 patients.

Eight patients received a second line of chemotherapy after progression. Three received paclitaxel, three received capecitabine, one received vinorelbine and one had holoxan-vepeside.

### Tumor response and survival

**Figure 1:**
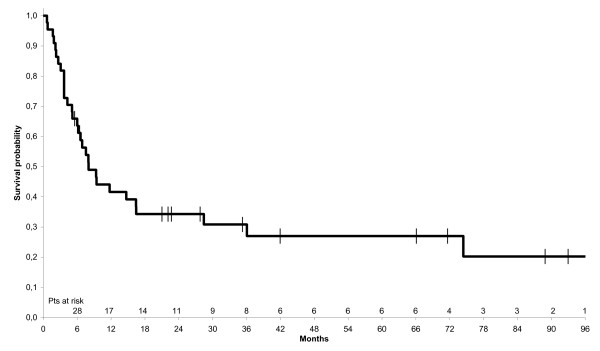
**Kaplan-Meier estimate of overall survival of all 44 patients with anaplastic thyroid carcinoma**.

**Figure 2:**
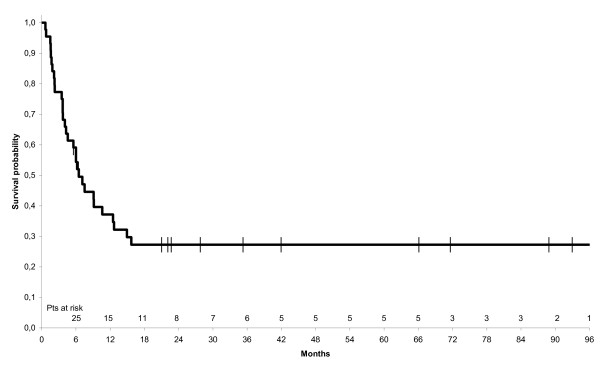
**Kaplan-Meier estimate of progression free survival of all 44 patients with anaplastic thyroid carcinoma**.

At the end of treatment, a complete response (CR) was observed in 14 patients (31.8%) and a partial response in 8 patients (18%). The median survival of these responders was 28.4 months; it was significantly different from that of progressive patients (50%) which was 5.1 months. With a median follow up of 7, 8 months (0,7, 160,6), 31 patients died and the median OS was 8 months (6, 16,5). The median PFS was 6, 5 months (4,3, 12,5) (Figures 1 and 2).

**Figure 3:**
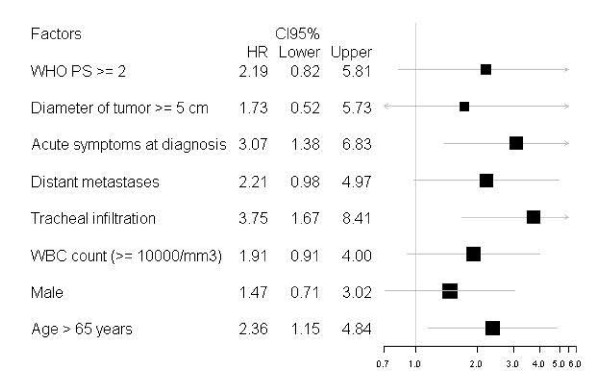
**Forest plot of hazard ratios for overall survival according to prognostic factors for 44 patients with anaplastic thyroid carcinoma**.

**Figure 4:**
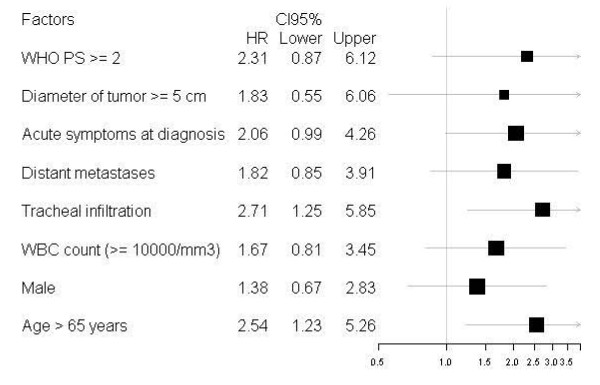
**Forest plot of hazard ratios for progression free survival according to prognostic factors for 44 patients with anaplastic thyroid carcinoma**.

Thirteen of the 44 treated patients are still alive, with a median overall survival (OS) of 8 months and a median progression-free survival (PFS) of 6.5 months. Median OS and PFS were significantly lower in the patients undergoing palliative surgery (debulking) than in those undergoing near-total or total thyroidectomy (3.7 vs. 14.7 months respectively for OS, 3.7 vs. 10.6 months respectively for PFS). No significant difference in median OS and PFS was seen between the patients with local stage ATC and those with loco-regional or metastatic stage disease. Significantly better median OS and PFS rates were reported in the patients who had 3 or less factors of poor prognosis than in those who had more than 3 (14.7 *vs*. 4 months respectively for OS, 9 vs. 4 months respectively for PFS). We analyzed individually clinicopathological factors relatives to patients and treatment as prognostic factors presented in Figure 3 and 4.

### Toxicity

The main toxicity was digestive, with 65% grade 3 and 4 nausea and vomiting during treatment.

Anemia and neutropenia were frequent among patients receiving doxorubicin and cisplatin.

Grade 3 and 4 neutropenia was observed in 22.7% of the patients, whereas grade 3 or 4 thrombocytopenia was observed in 2.3%. The hematological toxicity was secondarily reduced by the systematic administration of G-CSF and erythropoietin. Weight loss was noted in 65% of the patients in spite of nutritional support during radiotherapy. Finally, one patient had reversible renal toxicity.

## Discussion

Anaplastic thyroid carcinoma is a highly lethal cancer with a spontaneous median survival of 4 to 12 months from the time of diagnosis [4]. It represents less than 2% of all thyroid cancers but still accounts for up to 40% of thyroid cancer mortality [9,10].

The characteristics of the patients in our study were similar to those described in the literature with an age between the sixth and seventh decades. There was a slight female to male predominance of 1.5 to 1. A large percentage of patients already had distant metastases at presentation. As in our study, the most common site of metastases reported in a 50-year review by McIver et al. [11] was the lung (42%), followed by bone (32%) and brain (9%). Although the identification of histological subtypes is an important part of the diagnosis of ATC, histological characteristics do not provide prognostic or predictive information regarding the patients' disease course or response to therapy. Because of a threat of local or metastatic spread, therapeutic options in ATC must combine local (cervical) and systemic treatments. Single modality therapy has been shown to have limited effect. Surgery or radiation, or chemotherapy alone rarely have significant impact on local-regional disease recurrence and overall survival. In the study by Veness et al. [12], 18 patients with ATC were treated at a single center, with 9 having single modality treatment (6 had radiation therapy, 3 had surgery, and 1 had chemotherapy). Four of the six patients receiving radiation therapy and two of the three patients undergoing surgery died of uncontrolled local disease. Because of the disappointing results from single modality therapies, multimodality therapy has progressively become the treatment of choice in ATC, with surgery being the cornerstone of patient management. Nearly all long-term survivors with ATC have had surgery as part of their treatment. In a retrospective review, Haigh et al. [13] describe five longterm survivors treated with surgery followed by RT/chemotherapy. More specifically, those patients who underwent a potentially curative resection (8/26) had a median survival of 43 months compared to 3 months for those with a palliative resection. In the study by Swaak-Kragten et al [14], patients who had R0/R1 resection and underwent protocol chemoradiation had a local complete response rate of 89%, compared to only 3% CR for patients receiving neither R0/R1 resection nor protocol radiation. Complete locoregional response resulted in longer median survival (7 months versus 3 months) and improved 1-year overall survival (32% versus 9%). Levendag et al. [15] has also described the achievement of a CR as an important goal for treatment. The above data confirm both the central role of surgery as part of multimodality therapy for ATC and the potential for long-term survival in a subset of patients. Unfortunately, as seen in our study and as reported by Haigh et al. [13], only a minority of patients can undergo microscopic tumor resection at the time of presentation.

The history of RT in the treatment of ATC has evolved from high dose conventional RT to hyperfractionated RT administered in combination with continuous chemotherapy. Since ATC is a rapidly dividing tumor, hyper-fractionated RT minimizes the opportunity for tumor cells to recover between treatments. Tennvall et al. [5] have reported improvement in local control with acceleration of hyperfractionated RT and concurrent chemotherapy. All 33 patients who achieved local control had total resection, but 30 had microscopic residual disease. This supports the hypothesis that RT can eradicate microscopic disease and improve survival when given in effective doses and fractionation schemes. Results published by Swaak-Kragten et al. [14] show that the total radiation dose appears to be an important prognosis factor; median overall survival was 5.4 months for patients treated with a total dose of > 40 Gy versus only 1.7 months for those receiving < 40 Gy. In the same study, the authors have added prophylactic lung irradiation (PLI) in combination with low-dose doxorubicin because of the very high percentage of patients developing lung metastases in the course of their disease. This treatment had not been described in the literature for ATC before. Interestingly, of the 11 M0 patients treated only two subsequently developed lung metastases; however, a significant benefit could not be demonstrated due to the small number of patients treated and the lack of a control arm. Some chemotherapy drugs have been tested to try to increase overall survival in metastatic ATC patients. Results of a randomized trial by Shimaoka et al. [16] comparing doxorubicin alone to doxorubicin and cisplatin in 39 patients with ATC have shown a response rate of 5% for the single agent compared to 18% for the combination therapy. The authors have concluded that combination therapy is superior in terms of efficacy but this efficacy does not balance the risk of increased toxicity. Ain et al. [17] have employed paclitaxel in a phase II trial and have demonstrated that treatment responders had a median survival of 32 weeks compared to only 7 weeks in non-responders. Multimodality treatments have demonstrated efficacy in improving local tumor control in some ATC patients and may increase survival in patients with good performance status and without renal or cardiac insufficiency or metastatic disease. Unfortunately, most of the studies published were retrospective or were based on very small numbers of patients [18,19]. In recent years, two important prospective trials have been published. First, Tennvall et al. [5] have treated 55 consecutive patients with three different treatment schedules, all containing surgery, hyperfractionated radiotherapy and chemotherapy.

Despite this aggressive approach, overall survival was poor with only 9% of patients surviving at 2 years. A second study by the IGR team, published by De Crevoisier et al. [6], has enrolled 30 patients (including 6 with lung metastases) treated with surgery, chemotherapy (doxorubicin and cisplatin) and hyperfractionated RT. Results described a survival of 46% at 1 year and 27% at 3 years. These results are similar to ours (40% and 25%, respectively) but higher than in most other studies [5,18,19]. When considering our patients and those from the IGR study we obtain a total of 74 French patients treated with the same protocol. The results of the 2 studies are equivalent, justifying the use of a combined treatment in different cases of AT, principally for non metastatic patients after thyroid surgery (either complete or incomplete), whatever the lymph node status (N0/N1). Secondly, for good PS metastatic patients with limited metastatic lesions we would recommend a multimodality treatment (chemoradiotherapy) and then total thyroidectomy whenever feasible. It clearly appears that the primary step of clinical management in ATC is to identify potentially curable patients with ≤3 factors of poor prognosis and to offer them multimodality treatment to improve their chances of survival. Regarding patients with more than 3 poor prognosis factors, we should avoid aggressive therapy and offer them best supportive care.

Regarding new antitumor therapies, ATC have poorly benefited from recent progresses. Some have been tested in ATC but results have been often disappointing. Targeted agents directed against VEGF-R like axitinib (AG-013736) or against BRAF and VEGF-R like sorafenib (Nexavar^®^) have been tested in phase II trials including patients with anaplastic thyroid carcinoma [20,21]. One response was obtained with axitinib and one stabilization with sorafenib.

Vascular disrupting agents as Zybrestat^® ^(fosbretabulin, combretastatin A4 phosphate (CA4P)) have been evaluated in ATC because of their antiangiogenic and cytotoxic activity. The promising results obtained *in vitro *foreshadowed the clinical efficacy of the drug [22]. at the 35^th ^European Society of Medical Oncology (ESMO) meeting, the preliminary results of a phase II-III study of Zybrestat^® ^in combination with chemotherapy in ATC were presented. In this study, 80 patients (out of the 180 planned) were randomized to receive Zybrestat^® ^(IV once a week) in combination with chemotherapy (paclitaxel-carboplatin every 3 weeks) versus chemotherapy alone. For the first time in ATC, the median overall survival time was increased by the association (5.1 months versus 4.1 months for patients receiving chemotherapy alone).

## Conclusions

Despite the ultimately dismal prognosis of ATC, multimodality treatment significantly improves local control and appears to achieve long-term survival in some patients. Hopefully, active ongoing research should identify systemic treatments to be used in combination with more conventional methods. A promising area of research includes potential targeted treatment modalities focusing on the genetic alterations found in ATC. For example, p53 gene mutations commonly occur in ATC and may explain the loss of control of cell proliferation [23] but no treatment based on this abnormality has yet emerged. Recently, a prospective randomized trial has shown that CA4P (a reversible tubulin-binding vascular-disrupting agent) induces a significant survival benefit when used in combination with carboplatin and paclitaxel [24]. Other areas of active research involve angiogenesis inhibitors and tyrosine kinase inhibitors [25].

## Competing interests

The authors declare that they have no competing interests.

## Authors' contributions

All authors collected data, reviewed the draft, provided comments or substantive revisions, and approved the final manuscript.

## Pre-publication history

The pre-publication history for this paper can be accessed here:

http://www.biomedcentral.com/1471-2407/11/469/prepub
